# Supplementary material for: A Comparative and Critical Analysis for In Vitro Cytotoxic Evaluation of Magneto-Crystalline Zinc Ferrite Nanoparticles Using MTT, Crystal Violet, LDH, and Apoptosis Assay
Source: Int J Mol Sci. 2023 Aug 16;24(16):12860. doi: 10.3390/ijms241612860 (PMC10454666; doi:10.3390/ijms241612860)
Supplement: Supplementary file 1 [file ijms-24-12860-s001.zip › ijms-2410365-supplementary.pdf]

SUPPLEMENTARY INFORMATION

Article

A Comparative and Critical Analysis for In Vitro Cytotoxic Evaluation of Magneto-Crystalline Zinc Ferrite Nanoparticles Using MTT, Crystal Violet, LDH, and Apoptosis Assay

Juan Luis de la Fuente-Jiménez [1], César Iván Rodríguez-Rivas [2], Irma Beatriz Mitre-Aguilar [3], Andrea Torres-Copado [1], Eric Alejandro García-López [3], José Herrera-Celis [4], María Goretti Arvizu-Espinosa [1], Marco Antonio Garza-Navarro [5], Luis Gerardo Arriaga [4], Janet Ledesma García [2], Domingo Ixcóatl García-Gutiérrez [5], Alejandro Zentella Dehesa [3,6,*], Ashutosh Sharma [1,*] and Goldie Oza [4]

1 Centre of Bioengineering, School of Engineering and Sciences, Tecnologico de Monterrey, Campus Queretaro, Av. Epigmenio González, No. 500, Fracc. San Pablo, Querétaro 76130, Mexico; juan.luis.delafuente@tec.mx (J.L.d.l.F.-J.); a01700238@tec.mx (A.T.-C.); goretti@tec.mx (M.G.A.-E.)

2 División de Investigación y Posgrado, Facultad de Ingeniería, Universidad Autónoma de Querétaro, Cerro de Las Campanas S/N, Querétaro 76010, Mexico; cesarmediuaq@gmail.com (C.I.R.-R.); janet.ledesma@uaq.mx (J.L.G.)

3 Unidad de Bioquímica, Instituto Nacional de Ciencias Médicas y Nutrición Salvador Zubirán, Vasco de Quiroga 15, Belisario Domínguez secc 16, Tlalpan, Mexico City 14080, Mexico; irma.mitrea@incmnsz.mx (I.B.M.-A.); garcialp007@gmail.com (E.A.G.-L.)

4 Centro de Investigación y Desarrollo Tecnológico en Electroquímica, Parque Tecnológico, Sanfandila, Pedro Escobedo, Querétaro 76703, Mexico; jlherrera@cideteq.mx (J.H.-C.); larriaga@cideteq.mx (L.G.A.); goza@cideteq.mx (G.O.)

5 Facultad de Ingeniería Mecánica y Eléctrica, FIME, Universidad Autónoma de Nuevo León, UANL, Av. Universidad S/N, Cd. Universitaria, San Nicolás de los Garza 66450, Mexico; marco.garzanr@uanl.edu.mx (M.A.G.-N.); domingo.garciagt@uanl.edu.mx (D.I.G.-G.)

6 Departamento de Medicina Genómica y Toxicología Ambiental, Programa Institucional de Cáncer de Mama, Instituto de Investigaciones Biomédicas IIB & Red de Apoyo a la Investigación, Universidad Nacional Autónoma de México, Ciudad Universitaria, Ave. Universidad 3000, Col. Copilco Universidad, Del. Coyoacán Ciudad de México, Mexico City 04510, Mexico

* Correspondence: azentell@iibiomedicas.unam.mx (A.Z.D.); asharma@tec.mx (A.S.)

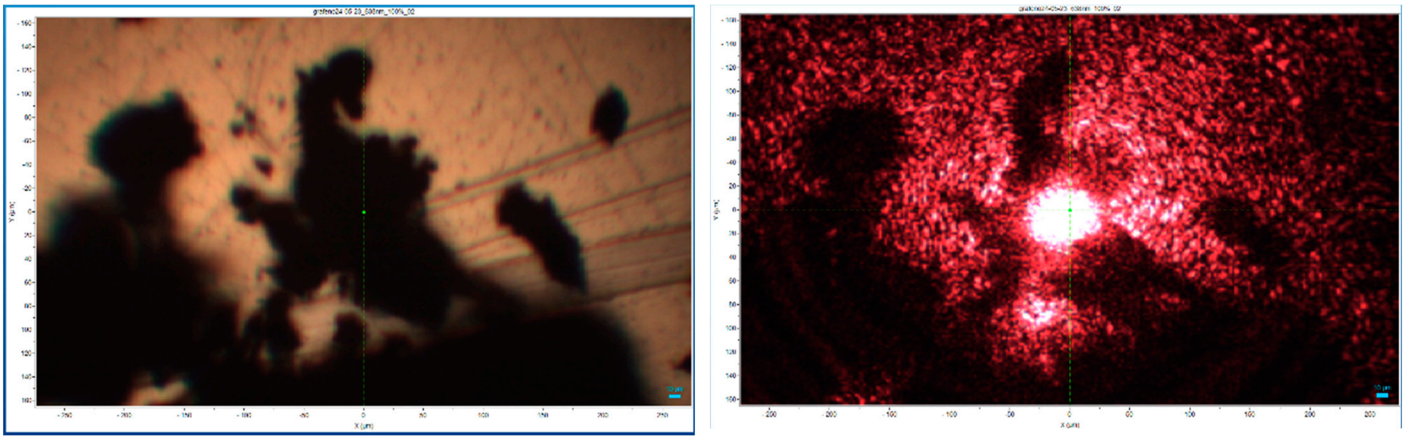

Figure S1. The microscopic image of ZFO NPs

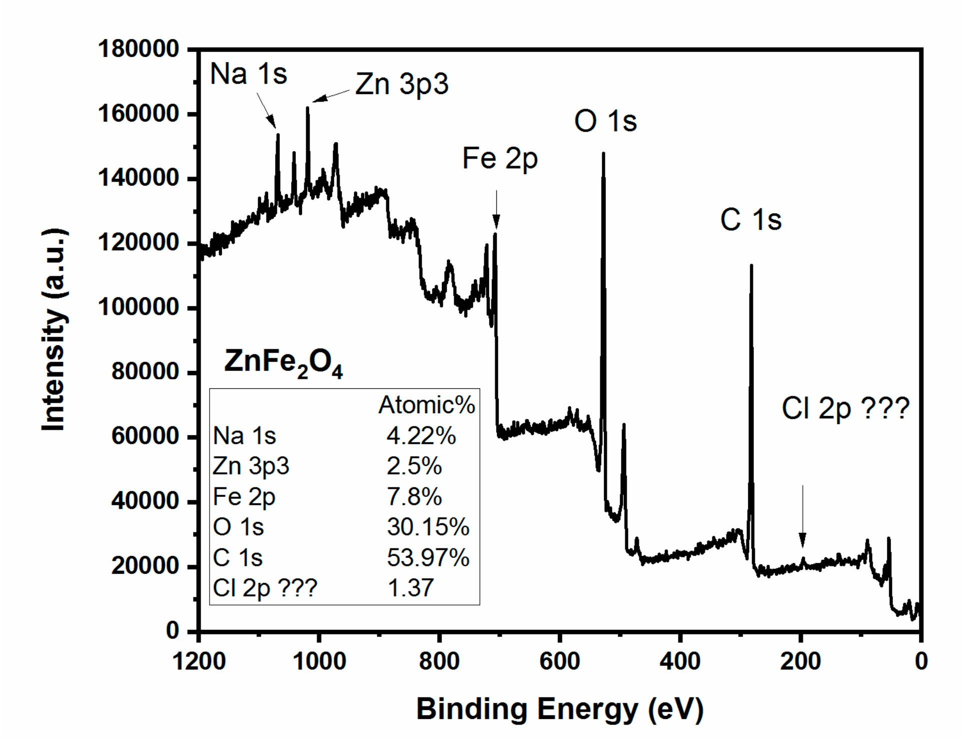

Figure S2. The complete survey scan of ZFO NPs

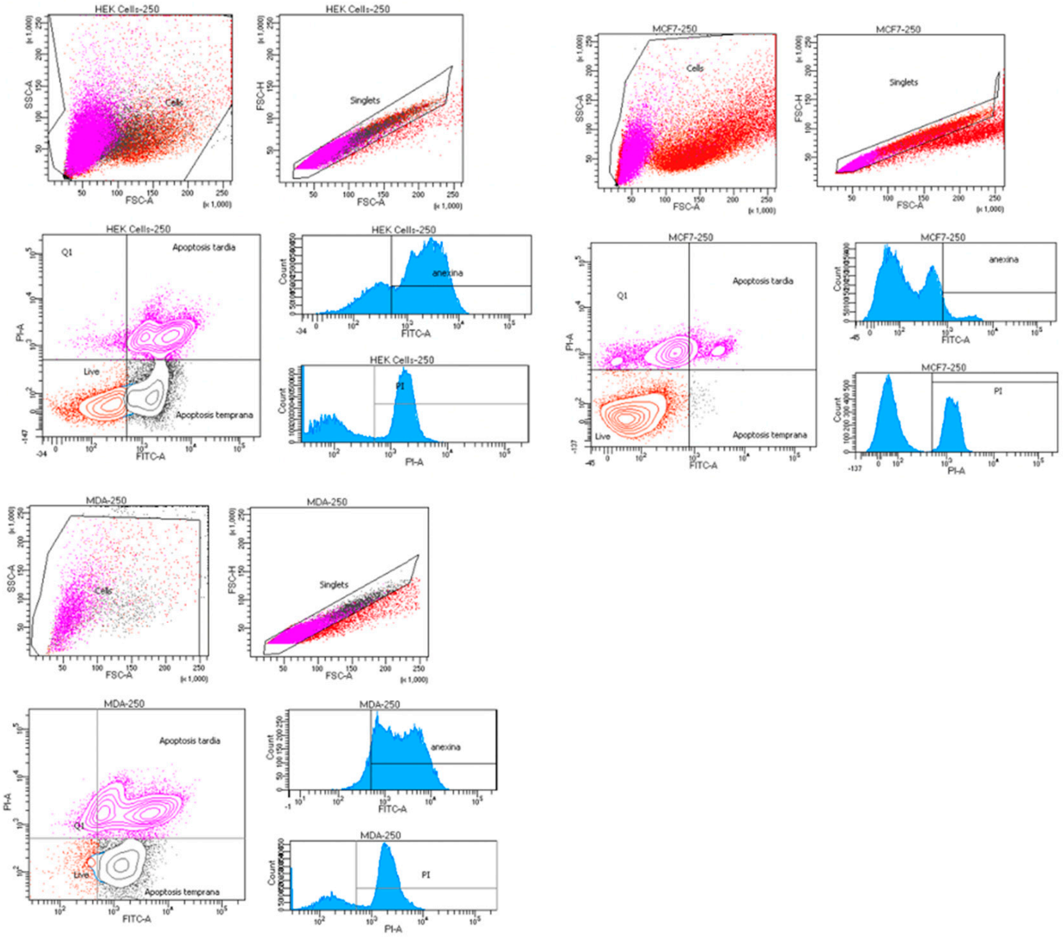

Figure S3. Representative information demonstrating the calibrated data and used gate between different cell lines (MDA-MB-231, MCF-7, and Hek-293) at the same exposed concentration (250 μg/mL) of zinc ferrite nanoparticles
